# “The Risk Seems Too High”: Thoughts and Feelings about COVID-19 Vaccination

**DOI:** 10.3390/ijerph18168690

**Published:** 2021-08-17

**Authors:** Ramey Moore, Don E. Willis, Sumit K. Shah, Rachel S. Purvis, Xochitl Shields, Pearl A. McElfish

**Affiliations:** 1 College of Medicine, University of Arkansas for Medical Sciences Northwest, 1125 N. College Avenue, Fayetteville, AR 72703, USA; rameymoore@uams.edu (R.M.); dewillis@uams.edu (D.E.W.); rspurvis@uams.edu (R.S.P.); 2 Office of Community Health and Research, University of Arkansas for Medical Sciences Northwest, 1125 N. College Avenue, Fayetteville, AR 72703, USA; sshah3@uams.edu (S.K.S.); xshields@uams.edu (X.S.)

**Keywords:** COVID-19 vaccine, vaccine hesitancy, vaccine attitudes, Increasing Vaccination Model

## Abstract

The purpose of this study was to describe the thoughts and feelings of individuals expressing concerns about the COVID-19 vaccine. A qualitative descriptive study was conducted in order to examine the thoughts and feelings of participants who are hesitant about the COVID-19 vaccine. Data were collected from 754 participants using an online instrument. Emergent themes included a lack of knowledge about the safety of the COVID-19 vaccine; concerns over the speed of development, testing, and approval of these vaccines; reluctance to be among the first vaccinated; concerns about the motivations of government actors, pharmaceutical companies, and others involved in producing the COVID-19 vaccine; and hesitancy about vaccines generally.

## 1. Introduction

Vaccines provide prophylaxis against a wide range of infectious diseases and are responsible for saving a significant number of lives over more than two centuries [1]. Promising preliminary results have also reported the effectiveness of therapeutic vaccines in the treatment of pre-cancerous and cancerous conditions [2]. Despite these achievements, vaccine hesitancy and vaccine refusal disrupt public health efforts in safeguarding populations against vaccine-preventable conditions [3].

The current COVID-19 pandemic has affected populations globally. The spread of COVID-19 infection and recurring surges in the number of new cases and deaths have caused catastrophic social and economic impacts around the world [4]. According to the Centers for Disease Control and Prevention (CDC), more than 32 million cases of COVID-19 have been recorded in the United States (US) [5]. During the pandemic, more than 80 governments restricted travelers from other countries [6], and nationwide lockdowns affected the quality of life for millions [7].

Vaccines designed to protect against COVID-19 were developed in an unprecedentedly rapid period of time, facilitated by advances in vaccine technologies [8]. Approximately 75–90% of the population needs to be immunized to reach population immunity; however, a substantial proportion of the global population is still hesitant to receive the COVID-19 vaccine [9,10].

Quantitative approaches have been used by various studies focused on examining COVID-19 vaccine attitudes and behaviors [11,12,13]. However, studies using qualitative approaches to understanding hesitancy towards the COVID-19 vaccine are still emerging and have focused on racial or ethnic or specific national populations [14,15]. This is the first qualitative study to analyze COVID-19 vaccine hesitancy among a broad general population in the US. The Increasing Vaccination Model (IVM) illustrates the role individuals’ thoughts and feelings play in shaping vaccine motivation and vaccination behaviors [16]. In order to fill this gap in knowledge about COVID-19 vaccine hesitancy, we conducted a qualitative study that leverages the IVM to examine the thoughts and feelings of participants who are hesitant about the COVID-19 vaccines.

## 2. Materials and Methods

### 2.1. Study Design and Sample

This study used a qualitative descriptive design [17]. This methodology focuses on summarizing respondent experiences and perceptions while also emphasizing the meanings attributed to their experiences [18,19]. Data were collected using an online survey with broad, open-ended questions to explore attitudes and lived experience related to the COVID-19 vaccine. Respondents were recruited from six primary care clinics located in different towns throughout the state of Arkansas between 30 October 2020 and 16 January 2021. Recruitment e-mails describing the study’s purpose and inclusion criteria were sent to 6092 e-mail addresses. A total of 876 responses to the survey were collected. Of those, 809 met the inclusion criteria; however, 21 provided no data past the eligibility screener, and an additional 34 duplicate records were removed. A total of 754 were determined to be non-duplicates who took part in the survey.

Research Electronic Data Capture (REDCap) was used to capture participant responses. REDCap is a widely used web-based software for working with survey data [20]. Inclusion criteria included being an adult (age ≥ 18) and living, working, and/or receiving health care in the state of Arkansas. Respondents reviewed study and consent information at the beginning of the online survey, and consent was documented in REDCap. A $20.00 gift card was provided as a participant incentive. Survey responses were stripped of identifying information prior to analysis. The study was approved by the Institutional Review Board at the University of Arkansas for Medical Sciences (UAMS) (IRB#261226).

In addition to the qualitative responses, questions from the Behavioral Risk Factor Surveillance System (BRFSS) were used to capture demographic information, including age, sex, income, race, and education [21]. Two vaccine attitude measures were used to assess general trust in vaccines [22] and vaccine hesitancy [23] specifically related to the COVID-19 vaccine. General vaccine trust was assessed by asking, “Overall how much do you trust vaccines?” Respondents could answer with “not at all”, “very little”, “somewhat”, “to a great extent”, and “completely”. Those who answered “to a great extent” or “completely” were coded as 1 to indicate a high level of trust. The other responses were coded as 0 to indicate lower levels of trust. COVID-19 vaccine hesitancy was measured by asking, “If a vaccine for COVID-19 were available today, what is the likelihood that you would get vaccinated?” Those who selected “unlikely” or “very unlikely” were coded as hesitant towards a COVID-19 vaccine. The survey questionnaire is included in Supplementary File 1.

### 2.2. Analytic Strategy

Demographic and vaccine attitude data were analyzed in order to document frequencies, percentages, and standard deviations. The IVM was used as an analytical framework to analyze the domain of participants’ “thoughts and feelings” related to the COVID-19 vaccination. The codebook was developed by the first author in collaboration with a second researcher on the qualitative analysis team using an iterative process grounded in a consensus model. The codebook was revised three times, and all open-ended responses were reviewed by two additional qualitative researchers. Analysis summaries and all coded segments were critically reviewed by the research team to ensure data, as well as illustrative excerpts from coded data, were extracted and categorized within the relevant thematic domain. Quotes were collated, and statements which best reflected emergent themes were chosen by consensus among the research team [24] and followed standard qualitative research practices that ensured coherence and data saturation [25]. The research team reviewed the data, codebook, and excerpts to ensure analytic rigor and reliability. All responses were coded using MAXQDA, a qualitative data analysis tool developed and supported by VERBI software [26]. Any divergence in the interpretation of coded segments was discussed by the research team and resolved with team-wide consensus. The research team has retained the punctuation and capitalization of the typed responses from respondents to ensure consistency with respondents’ actual words. Respondents provided over 300 responses to open-ended questions; therefore, only the most representative quotes are presented. Quotes are organized within the theme that the quote best represents; however, participants’ comments were multi-faceted, and they often discussed multiple, interrelated experiences within the same sentence.

## 3. Results

Table 1 provides descriptive characteristics of the study sample. Most respondents were between 30 and 49 years of age (42.66%), self-identified as women (70.42%), identified their race and ethnicity as non-Hispanic White (70.41%), and made less than $50,000 per year (66.77%). The present study uses a non-probability sample that is not fully representative of the population of Arkansas as a whole. The sample is diverse with regard to race and ethnicity but includes more women and individuals with a higher income than the median household income in Arkansas [27]. Table 1 also provides measures of general vaccine trust and hesitancy related to COVID-19 vaccines specifically. The majority of participants indicated a high trust for vaccines (56.48%) and were not hesitant regarding the COVID-19 vaccine (61.25%).

Qualitative responses indicating concerns regarding the COVID-19 vaccine were analyzed with the IVM domain of thoughts and feelings serving as an a priori code, with emergent themes arising within this overarching code category. Emergent subthemes within the IVM domain of thoughts and feelings are: not enough knowledge/information to judge the safety of the COVID-19 vaccine, the process for developing the COVID-19 vaccine was rushed and therefore cannot be trusted, do not want to be among the first to receive the COVID-19 vaccination, a lack of trust in those developing the COVID-19 vaccination, and a lack of trust in vaccines in general. The frequency of emergent themes is presented in Table 2. We describe each of these emergent themes below. 

### 3.1. Not Enough Knowledge/Information to Judge the Safety of the COVID-19 Vaccine

Respondents reported distrust because of the lack of information available to judge the safety of the COVID-19 vaccine. One response succinctly stated, “It’s very scary because not enough is known about this vaccine. It’s not had time to prove itself or to prove how safe it is. Does it really work and for how long” (#634). Another respondent stated that she “would not put my trust in something that hadn’t stood the test of time as other vaccines have” (#192). One respondent wrote that she is “unsure [the COVID-19 vaccine] is safe,” (#566). Another respondent discussed his lack of security related to safety from another direction; he stated, “safety assurance is absolutely necessary” (#117).

Respondents reported that more information about the safety of the COVID-19 vaccine would be needed before they would be comfortable with a vaccine. One respondent stated, “I will only get vaccinated against COVID19 if and when it has been thoroughly tested and proven to be safe and effective” (#98). Another respondent stated that she was hesitant and “would not rush to receive a vaccine until it has had more time to be tested for safety” (#217). Linked to her safety-related hesitancy, she added that she “would have to know more about a COVID-19 vaccine before I would take it” (#217). Another respondent stated that she “would like to know about the real safety of [a COVID-19 vaccine]” (#402).

### 3.2. The Process for Developing the COVID-19 Vaccine Was Rushed and Therefore Cannot Be Trusted

The rapid COVID-19 vaccine approval process was a major theme reported. The speed of the emergency use authorization (EUA) process was often cited as a salient factor leading to distrust of the COVID-19 vaccine. Respondents frequently stated that “It was made way too fast for me to trust it at all” (#742), and “scared…because [the COVID-19 vaccine] was pushed through fast.” Another respondent echoed this concern: “I worry about rushed clinical trials leading to an unsafe vaccine” (#59), specifically citing the clinical trial period as the most concerning feature of the approval process. Other respondents focused on the process as a whole: “I think that this vaccine needs to follow all the clinical trials and protocols that all vaccines follow and not be rushed through or cut corners. Seems to me this is the case which is why I will not be taking it” (#78). Other respondents stated, “they are rushing it seems” (#85) and described the COVID-19 process as a “race to release” (#311).

### 3.3. Do Not Want to Be among the First to Receive the COVID-19 Vaccination

Respondents frequently described their hesitancy and desire to wait and observe the effects of the vaccination on others. This perspective was succinctly documented by one respondent: “I would like to wait a bit longer to see if any long-term side effects appear before getting the vaccine” (#746). Another respondent connected being in a higher risk age category as justification for her “wait and see” approach. This respondent stated she “will not be vaccinated when they first come out. I am 79 years old. I will wait a few months to see how it goes” (#226). Another respondent echoed this sentiment: “Would like more time. I do not want to be a guinea pig for I am too old” (#238). Another respondent, self-identified as a vaccine coordinator and nurse, articulated her concerns: “I am the vaccine coordinator/nurse for my clinic, so I am a great promoter of vaccinations. However, unless I was required by my employer, I would not take the COVID vaccination at immediate release” (#311).

### 3.4. A Lack of Trust in Those Developing the COVID-19 Vaccination

Respondents reported distrust in the people, politicians, and for-profit companies responsible for the development of the COVID-19 vaccination. One respondent described distrust related to “the people developing this [COVID-19] vaccine,” with this respondent noting that individuals involved with the COVID-19 vaccine development were “untrustworthy” (#149). Another respondent stated that for-profit COVID-19 vaccine development negatively impacted her trust: “Trusting a vaccine that a company will profit from greatly causes a small about of doubt for me” (#534). Another respondent described his distrust with the motivations and trustworthiness of those working to develop vaccines. This respondent stated, “I don’t trust it, because the people developing this vaccine are untrustworthy” (#149). Other respondents cited the highly politicized approval process in their comments: “With the current administration handling of COVID-19 I don’t trust the efficacy of a vaccine” (#85). Another respondent also identified the political process of vaccine development as affecting her decision regarding vaccinating her children: “This vaccination has become such a political topic. I would not vaccinate myself or my children until it had been on the market longer” (#311).

### 3.5. A Lack of Trust in Vaccines in General

Other respondents noted a general distrust of vaccines. Respondents drew on personal experiences with flu vaccines to describe distrust of vaccines in general. One stated: “I have had flu shots in the 42 years of nursing and I always got sick, the times I did not get a flu shot I was fine. So no I wont take a shot” (#338). Another respondent also linked experiences with the flu vaccines to general distrust of vaccines. She stated that she “[looks] at a COVID vaccine the same way [she does] a flu vaccine” and that she has “never gotten the [flu] shot or the flu” (#185). Fear of vaccinations in general was also reported. One respondent noted that he wanted “the option to walk away [from a vaccination] without being made to feel bad because I was afraid” and he closed his comment stating “please, no forced injections” (#187).

Another respondent focused her comments on pharmaceutical companies, expressing a blanket negative opinion of vaccines. She expressed disbelief that any individual would trust “JOHNSON & LYING JOHNSON & Pfizer” in particular and stated her suspicion that vaccines are deadly and supported by “lies” (#601). One respondent described her views of vaccines, referring broadly to vaccines as “eugenics on steroids” (#456). This respondent used sarcasm to describe salient aspects of vaccination, stating, “Vaccines, so safe they need to be force mandated at gunpoint. Vaccines, so safe the manufactures are not held accountable for the damages their vaccines cause. Vaccines, so safe they have to have a secret vaccine court that has paid out over $5 billion for vaccine damages. How are unvaccinated people a threat if vaccines worked???” (#456).

## 4. Discussion

Vaccination has become an increasingly important topic for global health and for public health responses to pandemics, such as the COVID-19 pandemic. This article used qualitative methods to understand and document the thoughts and feelings of respondents regarding the COVID-19 vaccine. Respondents described their perspectives in their own words, allowing a detailed analysis of salient features of vaccination beliefs of individuals who expressed concerns about the COVID-19 vaccine. Respondents articulated concern with a lack of knowledge about the COVID-19 vaccine, particularly related to vaccine safety. This finding is consistent with emerging literature specific to COVID-19 [28,29] and is also consistent with research on vaccines for other preventable diseases [30,31]. The qualitative analysis in this paper provided more of a nuanced understanding that adds to the prior literature.

Respondents expressed concern over the speed of the development, testing, and approval process. Our findings are consistent with the growing literature on vaccine hesitancy, in which the approval process for vaccines is an important contributing factor for the trust or mistrust in vaccines [32,33,34], and this article makes an important contribution as the first qualitative study highlighting participant concerns related to the approval process for COVID-19 vaccines in their own words. 

Respondents described their reluctance to be among the first recipients of the COVID-19 vaccine. Although the desire to not be among the first vaccinated likely involves multiple factors, respondents expressed their reticence, linking safety concerns to concerns related to the clinical testing and approval process. While other research has demonstrated hesitancy related to being part of the initial vaccine deployment [35], this is the first qualitative article to document this in the participants’ own words.

Respondents reported concerns about the people, governmental entities, and corporations involved in developing, authorizing, and distributing a COVID-19 vaccine. They described their suspicion that the political and economic systems involved in the development would affect the safety and/or efficacy of the COVID-19 vaccine. Distrust in public institutions is also well-described in the literature, with strong associations between the distrust of organizations, such as the CDC or the World Health Organization (WHO), and vaccine hesitancy [29,36]. Our findings are consistent with research demonstrating that the political landscape for vaccinations is critical for its impact on individual thoughts and feelings, as well as vaccine behaviors [32,37]. Our findings are consistent with research about the effects of the politicization of health issues generally [38], as well as newly-published findings regarding the role of politics in the COVID-19 pandemic [39]. Our findings contribute to burgeoning literature that links vaccine attitudes to the social and political aspects of the vaccine authorization and approval processes and is one of the first qualitative articles to do so.

Finally, some respondents described concerns with the COVID-19 vaccine as part of their concerns with vaccines in general. Respondents who expressed this concern focused on personal experiences with other vaccinations, such as the influenza vaccination. The experience of individuals or of people in close social proximity is suggested in prior research on vaccine hesitancy drivers [35]. These findings, alongside emerging literature on vaccine hesitancy in minority communities, demonstrate that reasoning by analogy with other vaccines is an important feature of individual thoughts and feelings and may be a major driver for vaccine-hesitant persons [35]. Other respondents focused on the economic, political, and juridico-legal context of vaccinations in general. These respondents placed particular emphasis on the profit motives of pharmaceutical companies and related to the fears of being forced to be vaccinated by government actors or through legislation. Distrust in corporate actors and pharmaceutical companies related to profit motives is also supported in the literature [40].

## 5. Limitations and Future Research

There are some limitations to this study. Digital data collection methodologies allow for a larger number of respondents and greater anonymity for respondents; however, this method for qualitative data capture does not allow for follow-up questions or clarifying probes. Digital survey methods may also introduce related biases, as potential participants may not have easy access to the survey or face other limitations on participation. Another limitation is that non-probabilistic samples may not be generalizable to other populations. Additional research is needed to explore the ways in which individual thoughts and feelings about vaccines are related to social networks and vaccine epistemologies. Further research is needed to understand how individuals identify trustworthy sources of information, how trusted messages are constructed, and what additional methods may be necessary in order to fill the information gap for vaccine-hesitant populations.

## 6. Conclusions

This study suggests that vaccine hesitancy related to the COVID-19 vaccine is both multi-dimensional and complex. Respondents viewed the COVID-19 vaccine as a risk, and vaccine hesitancy was linked to social, political, and economic processes related to vaccine production. The stakes are high for understanding the nuances of vaccine hesitancy, as a poor and disproportionate vaccine uptake will worsen the existing social and economic challenges, widen the health disparities gap, and diminish the efforts of equitable vaccine allocation. The results from this study are important for COVID-19 vaccination efforts and will improve efforts to understand the hesitancy or refusal of current and future vaccines, decreasing the burden of communicable diseases.

## Figures and Tables

**Table 1:** Sociodemographics, general vaccine trust, and COVID-19 vaccine hesitancy.

	Frequency	Percentage (%)	S.D.	Range
Age	754		16.31	18.2–90.6
18–29	112	15.36		
30–49	311	42.66		
50–64	177	24.28		
65+	129	17.70		
Sex	752		0.45	0–1
Women	531	70.42		
Men	221	29.31		
Race/Ethnicity	747		0.65	1–4
Black/African American	128	17.14		
White	526	70.41		
Other Race or Multiracial	61	8.17		
Hispanic/Latinx	32	4.28		
Income	620		1.19	1–4
<$25K	281	45.32		
$25K < $50K	133	21.45		
$50K < $75K	72	11.61		
>$75K	134	21.61		
Education	748		0.80	1–3
High School or Less	212	28.34		
Some College	265	35.43		
Four-Year Degree	271	36.23		
Rural–Urban Commuting Area (RUCA)	522		0.44	0–1
Non-Metro	142	27.20		
Metro	380	72.80		
General Vaccine Trust	694		0.49	0–1
Low Trust	302	43.52		
High Trust	392	56.48		
COVID-19 Vaccine Hesitancy	720		0.49	0–1
Hesitant	279	38.75		
Not Hesitant	441	61.25		

**Table 2:** Qualitative themes and percentages of appearance in coded segments.

Qualitative Themes	Percentage (%)
Not enough knowledge/information to judge the safety of the COVID-19 vaccine	40
The process for developing the COVID-19 vaccine was rushed and therefore cannot be trusted	34
Do not want to be among the first to receive the COVID-19 vaccination	12
A lack of trust in those developing the COVID-19 vaccination	13
A lack of trust in vaccines in general	4

## Data Availability

The deidentified data underlying the results presented in this study may be made available upon request from the corresponding author Dr. Pearl A. McElfish, at pamelfish@uams.edu. The data are not publicly available in accordance with funding requirements and participant privacy.

## Supplementary Materials

The Supplementary Materials are available online at https://www.mdpi.com/article/10.3390/ijerph18168690/s1.
