# Supplementary material for: “The Risk Seems Too High”: Thoughts and Feelings about COVID-19 Vaccination
Source: Int J Environ Res Public Health. 2021 Aug 17;18(16):8690. doi: 10.3390/ijerph18168690 (PMC8394920; doi:10.3390/ijerph18168690)
Supplement: Supplementary file 1 [file ijerph-18-08690-s001.zip › ijerph-1285541-supplementary.pdf]

Title: Impact of COVID-19 on Persons with and without Type 2 Diabetes
Principle Investigator: Pearl McElfish

S1. Survey Questionnaire

Eligibility

1. What is your date of birth? ____________________
   - ☐ Don't know/Not sure
     - If you are not sure, what do you estimate your age to be? __________________
   - ☐ Prefer not to answer
2. Do you live in Arkansas?
   - ☐ Yes
   - ☐ No
     Do you work or receive healthcare in Arkansas?
     - ☐ Yes
     - ☐ No
3. What county do you live in? _______________________(drop down list of counties with "other" option)
   If selected Other, please provide county and state where you live: ____________________

For those persons who do not meet the eligibility criteria:
Only those who are 1) 18 years of age or older, 2) live in Arkansas, or 3) work or receive health care in Arkansas, are eligible to participate in this study. If you do not meet those criteria, you are not eligible to participate in this study and you will not be compensated for completing this survey.

Background/Demographics

4. Date: ________________
5. What is your sex?
   - ☐ Male
   - ☐ Female
   - ☐ Prefer not to answer
6. Are you of Hispanic or Latino/a origin? (Select only ONE)
   - ☐ Yes, Hispanic or Latino
   - ☐ No, Not Hispanic or Latino
   - ☐ Don't know
   - ☐ Prefer not to answer
7. What is your race? (Select ALL that apply)
   - ☐ American Indian or Alaska Native
   - ☐ Asian
   - ☐ Black or African American
   - ☐ Native Hawaiian or Other Pacific Islander
   - ☐ White
   - ☐ Other: ____
   - ☐ Don't know
   - ☐ Prefer not to answer
8. How well do you speak English?
   - ☐ Very well
   - ☐ Well
   - ☐ Not well
   - ☐ Not at all
   - ☐ Don't Know/Not Sure
   - ☐ Prefer not to answer

9. Are you...?
   - ☐ Single
   - ☐ Married
   - ☐ Divorced/Separated
   - ☐ Widowed
   - ☐ A member of an unmarried couple
   - ☐ Don't Know/Not Sure
   - ☐ Prefer not to answer
10. What is the highest grade or year of school you completed?
   - ☐ Never attended school or only attended kindergarten
   - ☐ Grades 1 through 8 (Elementary)
   - ☐ Grades 9 through 11 (Some high school)
   - ☐ Grade 12 or GED (High school graduate)
   - ☐ College 1 year to 3 years (Some college or technical school)
   - ☐ College 4 years or more (College graduate)
   - ☐ Don't Know/Not Sure
   - ☐ Prefer not to answer

COVID-19 Screening

11. Have you had the nose swab test for the virus that causes COVID-19?
   - ☐ No, I never tried to get tested
   - ☐ No, I tried to get tested but was not able to
   - ☐ Yes
   - ☐ Don't know/Not sure
   - ☐ Prefer not to answer
12. We would like to understand where to make COVID testing most accessible to you and your community. Where would you prefer to have COVID-testing available? (select up to 3)
   - ☐ Church/Faith-based organization
   - ☐ Clinic in a hospital
   - ☐ Standalone clinic
   - ☐ Pharmacy
   - ☐ Community-based organization in my neighborhood
   - ☐ School in my neighborhood
   - ☐ Through health worker that comes to my home
   - ☐ Arkansas Department of Health
   - ☐ Worksite
   - ☐ Other (specify):
     Other location I would prefer to have COVID-19 testing available: _____________
   - ☐ Don't know/Not sure
   - ☐ Prefer not to answer
13. Would you prefer drive-through or walk-up testing at the locations you selected?
   - ☐ Drive-through
   - ☐ Walk-up
   - ☐ No preference
14. Is there anything else you would like to tell us about COVID-19 testing? __________________
   ____

Vaccinations

15. Overall how much do you trust vaccines?

- ☐ Not at all
- ☐ Very little
- ☐ Somewhat
- ☐ To a great extent
- ☐ Completely
- ☐ Don't know/Not sure
- ☐ Prefer not to answer

16. If a vaccine for COVID-19 were available today, what is the likelihood that you would get vaccinated?
    - ☐ Extremely likely
    - ☐ Somewhat likely
    - ☐ Unlikely
    - ☐ Very unlikely
    - ☐ Don't know/Not sure
    - ☐ Prefer not to answer
17. Would you be willing to participate in a COVID-19 vaccine clinical study or trial?
    - ☐ Yes
    - ☐ Maybe
    - ☐ No
    - ☐ Don't know/Not sure
    - ☐ Prefer not to answer
18. Where would you prefer to receive a vaccine for COVID-19? ___________________________
19. Is there anything else you would like to tell us about COVID-19 vaccines? ________________

Trusted sources of COVID-19 information

	To a Great Extent	Somewhat	Very Little	Not at All	Don't know/Not sure	Prefer not to answer
20. Local television news						
21. National television news						
22. Daily or weekly newspapers						
23. Conversations with family and friends						
24. Conversations with colleagues						
25. My pastor						
26. My primary care provider						
27. Websites or online news pages						
28. Social media (e.g. Facebook, Twitter, YouTube, WhatsApp)						
29. Radio stations						
30. U.S. Department of Health (includes CDC, NIH, and other federal health agencies)						
31. Arkansas Department of Health						
32. University of Arkansas for Medical Sciences (UAMS)						
33. Other medical institution press releases						
34. Celebrities and social media influencers						

35. Other sources (please specify):___________						

36. Is there anything else you would like to tell us about trusted sources of information about COVID-19?________________________________

Impacts of COVID-19 on Daily Life

The COVID-19 outbreak started in early 2020. For the purposes of the survey, we consider the time before the COVID-19 outbreak as last year, 2019. We consider the time after / since the COVID-19 outbreak as being this year, 2020.

37. Please tell us how COVID-19 has affected you daily life: ____________________
38. What have been your greatest sources of stress from the COVID-19 outbreak? (Select ALL that apply)
    - ☐ Health concerns
    - ☐ Financial concerns
    - ☐ Impact on work
    - ☐ Impact on your child
    - ☐ Impact on your community
    - ☐ Impact on family members
    - ☐ Access to food
    - ☐ Access to baby supplies (e.g., formula, diapers, wipes)
    - ☐ Access to personal care products or household supplies
    - ☐ Access to medical care, including mental health care
    - ☐ Access to medical supplies (e.g., prescriptions, testing supplies, etc.)
    - ☐ Social distancing or being quarantined
    - ☐ Other (please specify):
      What other source(s) of stress have you experienced during the COVID-19 outbreak? ________________________________
    - ☐ I am not stressed about the COVID-19 outbreak
    - ☐ Don't know/Not sure
    - ☐ Prefer not to answer

Since the COVID-19 outbreak began, what has changed for you or your family?

	Yes	No	Does not apply	Don't know/ Not sure	Prefer not to answer
39. More quality time with family or friends in person or from a distance (e.g., on the phone, Email, social media, video conferencing, online gaming).					
40. Improved relationships with family or friends.					
41. Spent *less* time on screens or devices outside of work hours (e.g., looking at phone, playing video games, watching TV).					

42. Is there anything else you would like to tell us about how COVID-19 has affected your daily life?________________________________
____

COVID-19 Attitudes/Fears/Beliefs

The COVID-19 outbreak started in early 2020. For the purposes of the survey, we consider the time before the COVID-19 outbreak as last year, 2019. We consider the time after / since the COVID-19 outbreak as being this year, 2020.

	No Chance	Some chance	High Chance	Don't know/Not sure	Prefer not to answer
43. What do you think your chances are of getting COVID-19?					
44. What do you think your chances are of dying from COVID-19?					

45. I know how to protect myself from COVID-19.
    - ☐ Not sure at all
    - ☐ Maybe/Not sure
    - ☐ Yes, completely sure
    - ☐ Prefer not to answer
46. For me, avoiding an infection with COVID-19 in the current situation is…
    - ☐ Extremely difficult
    - ☐ Difficult
    - ☐ Easy
    - ☐ Extremely easy
    - ☐ Don't know/Not sure
    - ☐ Prefer not to answer

47. Why do you feel that avoiding an infection with COVID-19 is [pipe in answer from previous question]?____

Because of COVID-19, I am concerned about…	To a Great Extent	Somewhat	Very Little	Not at All	Don't know/Not sure	Prefer not to answer
48. being infected myself						
49. the impact of being infected on my health, including dying						
50. being isolated from other people						
51. losing my job / family income						
52. losing my / family's savings						

53. not having enough money for food and/or rent						
54. infecting other people I live with						
55. a person I live with being infected						
56. a family member with whom I do not share my home being infected						
57. a friend with whom I do not share my home being infected						
58. infecting other people in the community						
59. there not being enough food left on shelves for people to eat						
60. my country going into an economic recession/depression						
61. how long it will take for things to go back to normal						

Housing

The COVID-19 outbreak started in early 2020. For the purposes of the survey, we consider the time before the COVID-19 outbreak as last year, 2019. We consider the time after / since the COVID-19 outbreak as being this year, 2020.

62. Including yourself, how many people currently live in your home?
    - ☐ Enter number of children (those under the age of 18 years):
    - ☐ Enter number of adults (those 18 years and older):
    - ☐ Don't know/Not sure
    - ☐ Prefer not to answer
63. Is the number of people currently living in your home more, less, or about the same as the number of people regularly living in your home before the COVID-19 outbreak began?
    - ☐ More than before
    - ☐ Less than before
    - ☐ About the same as before
    - ☐ Don't know/Not sure
    - ☐ Prefer not to answer
64. Please check all of the following that have happened to you since the COVID-19 outbreak
    - ☐ I have moved into a friend or family member's household
    - ☐ A friend or family member(s) have moved into my household
    - ☐ I became homeless (e.g., lived in a shelter, outside, or in a car)
    - ☐ None of these
    - ☐ Don't know/Not sure
    - ☐ Prefer not to answer
65. In the past 12 months, how many times have you or your family moved from one home to another? ____________________

Employment and Income

The COVID-19 outbreak started in early 2020. For the purposes of the survey, we consider the time before the COVID-19 outbreak as last year, 2019. We consider the time after / since the COVID-19 outbreak as being this year, 2020.

66. Prior to the COVID-19 outbreak, how would you describe your employment status?
    - ☐ Employed for wages (35+ hours per week)
    - ☐ Employed for wages part time (34 hours per week or less)
    - ☐ Self-employed
    - ☐ Out of work for 1 year or more
    - ☐ Out of work for less than 1 year
    - ☐ Taking care of your family and home and do not work for wages
    - ☐ A non-working student
    - ☐ Retired
    - ☐ Unable to work
    - ☐ Prefer not to answer
67. Has your employment status changed since the COVID-19 outbreak?
    - ☐ Yes
    - ☐ No
    - ☐ Prefer not to answer
68. (If "Yes") Are you currently…?
    - ☐ Employed for wages (35+ hours per week)
    - ☐ Employed for wages part time (34 hours per week or less)
    - ☐ Self-employed
    - ☐ Out of work for 1 year or more
    - ☐ Out of work for less than 1 year
    - ☐ Taking care of your family and home and do not work for wages
    - ☐ A non-working student
    - ☐ Retired
    - ☐ Unable to work
    - ☐ Prefer not to answer
69. Prior to the COVID-19 outbreak, how would you best describe your primary job sector?
    - ☐ Health care worker (e.g., doctor, nurse, nursing aide) or first responder
    - ☐ Work in a clinic or hospital but not as a health care worker
    - ☐ Work in a food processing plant
    - ☐ Student
    - ☐ Faith
    - ☐ School/education system (as employee not student)
    - ☐ Professional (physical/earth science/engineering professional, business/sales and marketing professional, software developer, legal, author, journalist, performing artist)
    - ☐ Manager (chief executive, administrative manager, production and sales, hospitality and retail manager)
    - ☐ Technician or associate professional (in field of engineering, business, legal, social, or information/communications)
    - ☐ Clerical support worker (office clerk, secretary, customer service clerk)
    - ☐ Service and sales worker (travel agent, cook, hair dresser/barber, retail sales, cashier)
    - ☐ Skilled agricultural, forestry and fishery worker
    - ☐ Craft and related trades worker (builder, machinist, electrician, printing)
    - ☐ Plant and machine operator and assembler (includes truck drivers)
    - ☐ Cleaning or maintenance worker occupations (cleaner, helper, agricultural laborer, transport laborer, street vendor, refuse worker)
    - ☐ Armed forces occupations (commissioned and non-commissioned)
    - ☐ Other (please specify):

      Other primary job sector: ____

- ☐ Don't know/Not sure
- ☐ Prefer not to answer

70. Do you have any form of paid sick leave from your job?
    - ☐ No
    - ☐ Yes
    - ☐ Don't know/Not sure
    - ☐ Prefer not to answer
71. In what ways has the COVID-19 outbreak affected your work? (Select ALL that apply)
    - ☐ I lost my job permanently
    - ☐ I lost my job temporarily
    - ☐ I got a new job
    - ☐ I reduced my work hours
    - ☐ I increased my work hours
    - ☐ My job put me at increased risk of getting COVID-19
    - ☐ I laid off employees
    - ☐ I did not have a paying job before the COVID-19 outbreak
    - ☐ None of these apply
    - ☐ Don't know/Not sure
    - ☐ Prefer not to answer
72. (If yes to currently employed for wages) Please check all of the following your workplace has done since the COVID-19 outbreak (Select ALL that apply)
    - ☐ Required employees to work remotely or from home
    - ☐ Suggested employees work remotely or from home
    - ☐ Required employees to wear face masks
    - ☐ Suggested employees wear face masks
    - ☐ Provided employees with face masks
    - ☐ Required social distancing at work
    - ☐ Installed barriers / shields to reduce contact between employees and others
    - ☐ Taken employee temperatures before entering workplace
    - ☐ Asked COVID-19 screening questions of employees before entering workplace
    - ☐ Was tested for COVID-19
    - ☐ I have not had a paying job since the COVID-19 outbreak
    - ☐ None of these
    - ☐ Don't know/Not sure
    - ☐ Prefer not to answer

73. Is your annual income from all sources—
    - ☐ Less than $10,000
    - ☐ $10,000 to less than $15,000
    - ☐ $15,000 to less than $20,000
    - ☐ $20,000 to less than $25,000
    - ☐ $25,000 to less than $35,000
    - ☐ $35,000 to less than $50,000
    - ☐ $50,000 to less than $75,000
    - ☐ $75,000 or more
    - ☐ Don't know/Not sure
    - ☐ Prefer not to answer
74. As a result of COVID-19, has your family income changed?
    - ☐ No
    - ☐ Yes, it decreased our income
    - ☐ Yes, it increased our income
    - ☐ Don't know/Not sure
    - ☐ Prefer not to answer
75. As a result from COVID-19, have you filed for unemployment benefits?
    - ☐ Yes, I've applied and have received it
    - ☐ Yes, I've applied but have NOT received it yet
    - ☐ No, but I plan to
    - ☐ No, because I don't know how to apply
    - ☐ No, I don't qualify for a payment
    - ☐ Don't know/Not sure
    - ☐ Prefer not to answer
76. Is there anything else you would like to tell us about how COVID-19 has affected your employment and/or income? ____

Health Status

The COVID-19 outbreak started in early 2020. For the purposes of the survey, we consider the time before the COVID-19 outbreak as last year, 2019. We consider the time after / since the COVID-19 outbreak as being this year, 2020.

77. Please tell us how COVID-19 has affected your physical and/or mental health:____________
78. Would you say that in general your health is…?
    - ☐ Excellent
    - ☐ Good
    - ☐ Fair
    - ☐ Poor
    - ☐ Don't Know/Not Sure
    - ☐ Prefer not to answer
79. Compared to the time before the COVID-19 outbreak, is your physical health status better, worse, or about the same?
    - ☐ Better
    - ☐ Worse
    - ☐ About the same
    - ☐ Don't know/Not sure
    - ☐ Prefer not to answer

80. Over the last two weeks how would you rate your mental health?
    - ☐ Excellent
    - ☐ Somewhat good
    - ☐ Average
    - ☐ Somewhat poor
    - ☐ Poor
    - ☐ Don't know/Not sure
    - ☐ Prefer not to answer

To your knowledge, has a doctor or healthcare professional ever told you that you have any of the following health conditions?

	Yes	No	Don't know/Not sure	Prefer not to answer
81. Any heart disease or history of heart attack or stroke				
82. High blood pressure				
83. Asthma				
84. Any chronic lung disease (e.g., chronic obstructive pulmonary disease, COPD/emphysema/chronic bronchitis)				
85. Type 1 diabetes (high blood sugar)				
86. Type 2 diabetes (high blood sugar)				
87. Active/current cancer				
88. Kidney disease				
89. Liver disease				
90. Any autoimmune disease (e.g., lupus, multiple sclerosis, rheumatoid arthritis, psoriasis, Crohn's disease, inflammatory bowel disease)				
91. Any depressive disorder (e.g., major depression)				
92. Any anxiety disorder (e.g., panic disorder, generalized anxiety disorder, post-traumatic stress disorder)				

93. Is there anything else you would like to tell us about how COVID-19 has affected your physical and/or mental health? ______________________________

Diabetes Module (If "Yes" to Type 1 or Type 2 diabetes on risk factors)

The COVID-19 outbreak started in early 2020. For the purposes of the survey, we consider the time before the COVID-19 outbreak as last year, 2019. We consider the time after / since the COVID-19 outbreak as being this year, 2020.

94. What was your most recent HbA1c (%)?______________________________

95. How different was this HbA1c reading from your average HbA1c reading before the COVID-19 outbreak?
   - Higher
   - Lower
   - About the same
   - Don’t know/Not sure
   - Prefer not to answer
96. How do you control your Type 2 diabetes now?
   - Insulin
   - Oral medication
   - Both insulin and oral medication
   - Neither insulin nor oral medication
   - Don’t know/Not sure
   - Prefer not to answer
97. Please tell us how COVID-19 has affected your management of Type 1 or Type 2 diabetes: _

The COVID-19 outbreak started in early 2020. For the purposes of the survey, we consider the time before the COVID-19 outbreak as last year, 2019. We consider the time after / since the COVID-19 outbreak as being this year, 2020.

BLOOD SUGAR TESTING										
98. Do you have a continuous glucose-monitor now?	Yes	No	Don’t know/ Not sure	Prefer not to answer						
99. Did you have a continuous glucose-monitor before the COVID-19 outbreak?	Yes	No	Don’t know/ Not sure	Prefer not to answer						
100. On how many of the last SEVEN DAYS did you test your blood sugar?	0	1	2	3	4	5	6	7	Don’t know/ Not sure	Prefer not to answer
101. Is this more, less or about the same as the number of days you tested your blood sugar in a typical week before the COVID-19 outbreak?	More	Less	About the same	Don’t know/ Not sure	Prefer not to answer					
102. How many times PER DAY does your health care provider recommend you test your blood sugar?	Number of times per day your health care provider recommends you test your blood sugar: ______________	Don’t know/ Not sure	Prefer not to answer							
103. On how many of the last SEVEN DAYS did you test your blood sugar at least ____ times per day?	0	1	2	3	4	5	6	7	Don’t know/ Not sure	Prefer not to answer
104. Is this more, less or about the same as the number of days you tested your blood sugar at least ____ times per day in a typical week before the COVID-19 outbreak?	More	Less	About the same	Don’t know/ Not sure	Prefer not to answer					
The COVID-19 outbreak started in early 2020. For the purposes of the survey, we consider the time before the COVID-19 outbreak as last year, 2019. We consider the time after / since the COVID-19 outbreak as being this year, 2020.										
FOOT CARE										
105. On how many of the last SEVEN DAYS did you check your feet?	0	1	2	3	4	5	6	7	Don’t know/ Not sure	Prefer not to answer

106. Is this more, less or about the same as the number of days you checked your feet in a typical week before the COVID-19 outbreak?	More	Less	About the same	Don't know/ Not sure	Prefer not to answer

107. On how many of the last SEVEN DAYS did you inspect the inside of your shoes?	0	1	2	3	4	5	6	7	Don't know/ Not sure	Prefer not to answer

108. Is this more, less or about the same as the number of days you inspected your shoes in a typical week before the COVID-19 outbreak?	More	Less	About the same	Don't know/ Not sure	Prefer not to answer

109. When was the last time you had an eye exam in which the pupils were dilated? This would have made you temporarily sensitive to bright light.
   - ☐ Within the past month (anytime less than 1 month ago)
   - ☐ Within the past year (more than 1 month ago but less than 12 months ago)
   - ☐ Within the past 2 years (more than 1 year ago but less than 2 years ago)
   - ☐ 2 or more years ago
   - ☐ Never
   - ☐ Don't know/Not sure
   - ☐ Prefer not to answer
110. Has the COVID-19 outbreak affected your level of access to the following? (Select ALL that apply)
   - ☐ Insulin
   - ☐ Needles
   - ☐ Blood glucose meters
   - ☐ Testing strips
   - ☐ Other diabetes medication
   - ☐ No issues to report
   - ☐ Other (please specify): ____
   - ☐ Don't know/Not sure
   - ☐ Prefer not to answer
111. Is there anything else you would like to tell us about how COVID-19 has affected your management of Type 1 or Type 2 diabetes? ____________________________________

Access to / Use of Health Services

The COVID-19 outbreak started in early 2020. For the purposes of the survey, we consider the time before the COVID-19 outbreak as last year, 2019. We consider the time after / since the COVID-19 outbreak as being this year, 2020.

112. Please tell us how has COVID-19 affected your overall health care (including ability to see a doctor or other health care provider and your ability to get needed medication):
113. Do you have one person you think of as your personal doctor or health care provider?
   - ☐ Yes, only one
   - ☐ More than one
   - ☐ No
   - ☐ Don't know/Not sure
   - ☐ Prefer not to answer
114. How many times did you see a doctor, nurse, or other health professional in 2019?
   - ☐ Enter the number of times
     _____ Number of times you saw a doctor, nurse, or other health professional in 2019
   - ☐ None
   - ☐ Don't know/Not sure

- ☐ Prefer not to answer

115. How confident are you filling out medical forms by yourself?
    - ☐ Extremely
    - ☐ Quite a bit
    - ☐ Somewhat
    - ☐ A little bit
    - ☐ Not at all
    - ☐ Don't know/Not sure
    - ☐ Prefer not to answer
116. In what ways has the COVID-19 outbreak affected your overall healthcare? (Select ALL that apply)
    - ☐ I did not go to healthcare appointments because I was concerned about entering my healthcare provider's office
    - ☐ My healthcare provider canceled an appointment
    - ☐ My healthcare provider changed to phone or online visits
    - ☐ My healthcare provider told me to self-isolate or quarantine
    - ☐ None of these apply
    - ☐ Don't know/Not sure
    - ☐ Prefer not to answer
117. How much difficulty do you have obtaining the medicine that you need because of the COVID-19 outbreak or social distancing rules?
    - ☐ None
    - ☐ Some
    - ☐ Much
    - ☐ Very difficult
    - ☐ Unable
    - ☐ Does not apply to me
    - ☐ Don't know/Not sure
    - ☐ Prefer not to answer
118. How much difficulty do you have with getting routine healthcare that you need because of the COVID-19 outbreak or social distancing rules?
    - ☐ None
    - ☐ Some
    - ☐ Much
    - ☐ Very difficult
    - ☐ Unable
    - ☐ Don't know/Not sure
    - ☐ Prefer not to answer

For each statement below, please indicate whether the COVID-19 outbreak has impacted you in the way described.

	Yes	No	Does not apply to me	Don't know/ Not sure	Prefer not to answer
119. Important medical procedure cancelled (e.g., surgery).					
120. Unable to access healthcare for a serious condition (e.g., dialysis, chemotherapy).					
121. Got less healthcare than usual (e.g., routine or preventive care appointments).					
122. Elderly or disabled family member not in your home unable to get the help they need.					

123. Do you currently have health insurance?
   - ☐ Yes
   - ☐ No
   - ☐ Don't know/Not sure
   - ☐ Prefer not to answer
124. How has your health insurance status changed since the COVID-19 outbreak? (Select only ONE)
   - ☐ I had health insurance ***before and during*** the COVID-19 outbreak
   - ☐ I had health insurance ***before*** the COVID-19 outbreak, but I have since lost it
   - ☐ I did ***not*** have health insurance ***before or during*** the COVID-19 outbreak
   - ☐ I did ***not*** have health insurance ***before*** the COVID-19 outbreak, but ***now*** I have it
   - ☐ Don't know/Not sure
   - ☐ Prefer not to answer

Telehealth Utilization

The COVID-19 outbreak started in early 2020. For the purposes of the survey, we consider the time before the COVID-19 outbreak as last year, 2019. We consider the time after / since the COVID-19 outbreak as being this year, 2020.

"Telehealth" uses video to allow you and your doctor to talk without being in the same room.

125. How have you used telehealth? (Select ALL that apply)
   - ☐ I used telehealth ***before*** the COVID-19 outbreak
   - ☐ I used telehealth ***during*** the COVID-19 outbreak
   - ☐ I have ***never*** used telehealth
   - ☐ Don't know/Not sure
   - ☐ Prefer not to answer
126. How likely are you to consider telehealth instead of appointments in person after the COVID-19 outbreak?
   - ☐ Very likely
   - ☐ Likely
   - ☐ Neither likely nor unlikely
   - ☐ Unlikely
   - ☐ Very unlikely
   - ☐ Don't know/Not sure
   - ☐ Prefer not to answer
127. Is there anything else you would like to tell us about how COVID-19 has affected your health care (including ability to see a doctor or other health care provider and your ability to get needed medication)? ________________________________

Internet Access

128. Do you or any member of your household own or use any of the following types of devices?

	Yes	No	Don't know/ Not sure	Prefer not to answer
a. Desktop or laptop				
b. Smartphone				
c. Tablet or other portable wireless device				
d. Some other type of computer (specify): What other type of computer do you or someone in your household use? ____________________				

129. Do you or any member of your household have access to the internet?
- ☐ Yes, by paying a cell phone company or Internet service provider
- ☐ Yes, without paying a cell phone company or Internet service provider
- ☐ No access to the Internet in this household
- ☐ Don't know/Not sure
- ☐ Prefer not to answer

<u>Diet and Physical Activity</u>

The COVID-19 outbreak started in early 2020. For the purposes of the survey, we consider the time before the COVID-19 outbreak as last year, 2019. We consider the time after / since the COVID-19 outbreak as being this year, 2020.

DIET

130. On how many of the last SEVEN DAYS did you eat five or more servings of fruits and vegetables?	0	1	2	3	4	5	6	7	Don't know/ Not sure	Prefer not to answer

131. Is this more, less or about the same as the number of days you ate five or more servings of fruits and vegetables in a typical week before the COVID-19 outbreak?	More	Less	About the same	Don't know/ Not sure	Prefer not to answer

132. Within the past 30 days, we worried whether our food would run out before we got money to buy more.
- ☐ Often true
- ☐ Sometimes true
- ☐ Never true
- ☐ Don't know/Not sure
- ☐ Prefer not to answer

133. Within the past 30 days, the food we bought just didn't last and we didn't have money to get more.
- ☐ Often true
- ☐ Sometimes true
- ☐ Never true
- ☐ Don't know/Not sure
- ☐ Prefer not to answer

EXERCISE

134. On how many of the last SEVEN DAYS did you participate in at least 30 minutes of a specific exercise session (such as swimming, walking specifically for exercise, biking, etc.) other than what you do around the house or as part of your work?	0	1	2	3	4	5	6	7	Don't know/ Not sure	Prefer not to answer

135. Is this more, less or about the same as the number of days you participated in at least 30 minutes of a specific exercise session (such as swimming, walking specifically for exercise, biking etc.) in a typical week before the COVID-19 outbreak?	More	Less	About the same	Don't know/ Not sure	Prefer not to answer

136. Is there anything else you would like to tell us about how COVID-19 has affected your diet and physical activity habits?

Research Subject Payment Voucher

Gift card in the amount of $20.00 provided for research participation.

First Name: ______________________________________

Last Name: ______________________________________

Email: ____

Street Address: ____

City: ____

State: ____

Zip Code: ____

Note: You must sign below to receive a gift card. Your gift card will be sent by the U.S. Postal Service and processing and mailing may take up to 30 business days.

Signature: ____

Thank you for your time.

Survey completion text: Thank you for completing the survey! Your responses have been received.
